# Ways to Success

**Published:** 1874-09

**Authors:** 


### WAYS TO SUCCESS.

OVER fifty years ago, a youth working on a farm, asked his father to give him money enough to buy a gun. The old man could not spare it, but the boy, nothing daunted, found an old piece of iron about the place, and in the course of time contrived to make a gun barrel out of it, with the very meagre facilities afforded by a country blacksmith's shop. He had not the materials to make a lock and stock, so he walked to the nearest town and "traded" for the necessary attachments, and was encouraged by the smith for having made so good a shooter; this gave him the ambition to make another, so he went to cutting out grindstones from the native rock to raise the money for gun materials; in a short time there was a considerable demand for guns of his make. During the French war with Prussia he was called upon to furnish guns for the army, and in less than eight months he made and delivered to the Government of France rifles of a particular pattern, costing five millions of dollars, which amount was duly paid. The same man furnishes rifles now for the United States, South America, Rome, Spain, Egypt and Japan. The farmer's boy who wanted a gun is now Elipholet Remington, of Ilion, New York. His manufactory covers four acres of ground, and he employs twelve hundred men. Not satisfied with this achievement, he has recently completed a Sewing Machine, which is reported to represent the latest and most perfect advance in the improvements of this important adjunct of domestic economy. This is the type of a boy who, when there is not a way, makes a way for himself. Many a youth would have sat down and "pouted," thinking over what a hard thing it was that he could not get a gun, with hard thoughts against the father for being so stingy. Not so with young Remington; he wanted a gun and was determined to have it; the very necessities of his situation stimulated him to the exercise and consequent development of the powers of planning and devising; in other words, of thinking for himself. And such are they, the world over, who achieve noted success. Those who think for themselves plan for themselves, and upon themselves lean. So it was with Fitch and Goodyear, and Howe. Their early history was the history of a struggle with privation and want, and imprisonment, and almost despair; and the immortal Morse must be added to the list, owing all to their patience and courage and indomitable persistence.

If young Remington had been supplied with a gun, he would have "gone a gunning," and fallen gradually into a kind of idle, loafing, aimless life, a burden to himself and a benefit to nobody. The very necessity of effort has been the making of many; while many more, who have had their wants gratified with the asking, have sunk into insignificance, and their name and memory have long since perished from the earth.

Some have been heard to express a wonder that the human family should be permitted by Infinite Benevolence to struggle against poverty and want. But as the human mind is constituted, it is better to struggle than to idle; better to work than to wait; better to lean on one's self than on another. It is the men who, as boys, struggled for a foothold in the world, who now wield the world's destinies. It is not the men who have inherited crowns, but those who have made crowns for themselves and have placed them on their own heads that have done the most in moulding the world's history.

Many a school child has marred its destiny, has been spoiled for all useful purposes in life in being helped too much in getting their lessons. Much may be done in teaching children to cherish self-reliance, determination and independence. More should be done than now is to inspire children with an ambition to find out ways of doing things for themselves. It is better to study out a rule in arithmetic or grammar; it would be a saving of time in the end, even if it took a month. The fundamental mischief of the public school system is, the children have not time to study out their lessons; they have not half an hour to give to any problem, and too often they must be shown how, or be disgraced with a discredit mark; small wonder is it that so many, especially girls, know almost nothing when they leave school; all they know is from the mechanical force of memory. The true object of going to school is not so much to become acquainted with things, to know things, but to learn how to think, how to devise, how to plan; how, if a thing cannot be done in one way, it may be accomplished in another; to spare no pains or labor or efforts to bring about what is desired, and to never give up until it is done, or is clearly impossible. This is the true way to make men and women worthy of their kind.

